# Bovine sperm selection procedure prior to cryopreservation for improvement of post-thawed semen quality and fertility

**DOI:** 10.1186/s40104-019-0395-9

**Published:** 2019-11-15

**Authors:** MariaPortia B. Nagata, Junki Egashira, Naoto Katafuchi, Kenji Endo, Kazuko Ogata, Kenichi Yamanaka, Tadayuki Yamanouchi, Hideo Matsuda, Yutaka Hashiyada, Kenichi Yamashita

**Affiliations:** 1 Advanced Manufacturing Research Institute, National Institute of Advanced Industrial Science and Technology (AIST), 807-1 Shuku-machi, Tosu, Saga, 841-0052 Japan; 2 Saga Prefectural Livestock Experiment Station, 23242-2 Yamauchi-machi, Miyano, Takeo, Saga, 849-2305 Japan; 3 Morinaga Dairy Service Co. Ltd., 1-159 Toyoharaotsu, Nasu-gun Nasu-machi, Tochigi, 329-3224 Japan; 4 National Livestock Breeding Center (NLBC), 1 Odakurahara, Odakura, Nishigo-mura, Nishishirakawa-gun, Fukushima, 961-8511 Japan; 5 Institute of Livestock and Grassland Science, National Agriculture and Food Research Organization (NARO), Ikenodai 2, Tsukuba, Ibaraki, 305-0901 Japan; 6 Faculty of Agriculture, Saga University, 1 Honjo-machi, Saga, 840-8502 Japan; 7 Ishikawa Prefectural University, 1-308 Suematsu, Nonoichi-shi, Ishikawa, 921-8836 Japan

**Keywords:** Cryopreservation, Fertility, Livestock, Rheotaxis, Spermatozoa, Thermotaxis

## Abstract

**Background:**

The application of cryopreservation and artificial insemination technology have contributed to the advancement of animal reproduction. However, a substantial proportion of spermatozoa undergoes alterations and loses their fertility during cryopreservation, rendering the frozen-thawed semen impractical for routine use. Cryopreservation is known to reduce sperm lifespan and fertility. Variation in cryosurvival of spermatozoa from different sires and even with the individual sire is common in artificial insemination (AI) centers. Our goal is to improve post-thawed semen quality by optimization of cryopreservation technique through sperm selection prior to cryopreservation process.

**Results:**

Our strategy of sperm selection based on rheotaxis and thermotaxis (SSRT) on macrosale in a rotating fluid flow demonstrated the ability to maintain the original pre-freezing structural integrity, viability and biological function related to fertilization competence. This strategy has a positive effect on the cryosurvival and fertilizing abilities of spermatozoa as supported by the improvement on pregnancy rate of Japanese Black heifers and Holstein repeat breeders. This technique protected further sublethal damage to bovine spermatozoa (higher % cryosurvival than the control) and resulted in the improvement of DNA integrity. Prefreeze selected spermatozoa demonstrated slower and controlled capacitation than unprocessed control which is thought to be related to sperm longevity and consequently to appropriate timing during *in vivo* fertilization.

**Conclusions:**

These results provide solid evidence that improvement of post-thawed semen quality by SSRT method is beneficial in terms of cryosurvival, longevity of post-thawed sperm, and optimization of *in vivo* fertilization, embryo development and calving as supported by the favorable results of field fertility study.

## Background

Following semen ejaculation is the irreversible aging of spermatozoa that will eventually lead to death. It is therefore, the aim of animal breeders to extend the life of the sperm cells. Semen cryopreservation is an important tool in artificial reproductive technologies (ART), propagation of animals with superior genetic traits, clinical medicine, and perpetuation of species. Cryopreserved semen samples have been widely used in the livestock breeding industry for artificial insemination (AI). Selection of genetically superior sires is essential in cattle production. In AI centers, it is a requirement to accurately select animals that possess high quality semen with sufficient post-thaw survival of cryopreserved semen referred to as freezability. However, some bulls with high genetic value produce semen sensitive to cryopreservation [1]. In Japan, only bulls that yield spermatozoa with post-thaw motility up to acceptable level would be considered for preservation. Therefore, collections that are classified as substandard and those that do not cryopreserve well are discarded and replaced by other bulls. Another challenge to semen cryopreservation is the observed seasonal variation in semen quality. There are observed seasonal effects on semen quality and seasonal reduction in sperm function [2]. Ejaculates collected during summer from heat-stressed bull were more sensitive to cryopreservation than those collected in the winter which might explain the decreased conception rate of cows in the summer [3]. Moreover, considerable variation in post thaw semen quality following cryopreservation among different bulls and even among ejaculates collected from the same bull exits. In our previous study, however, we identified the fertile subpopulation based on the kinetic and trajectory patterns, and correlated this subpopulation to field fertility and successful livebirths [4]. Sperm sorting resulted in subpopulation of sperm with improved fertility parameters and such subpopulation is believed to exist within the same sperm samples that resulted in successful pregnancy. However, microfluidic chip sorted sperm will be limited to regional application (to AI centers with appropriate equipment e.g. Microscope) unless sorted sperm can be frozen efficaciously. It is believed that isolation of such fertile subpopulation with desirable characteristics done by bulk sorting could afford greater flexibility in livestock management. Therefore, there is a need for a technology that can mitigate the detrimental effects of dead and damaged sperm by removal of this subpopulation and isolation of motile and fertile subpopulation regardless of season, which will consequently result to enrichment of the semen doses with viable, highly motile, and fertile spermatozoa that will in turn improve pregnancy rate. Moreover, non-viable spermatozoa due to programmed death or apoptosis, more likely prior to ejaculation, and necrosis, resulting from severe cell stress due to an external source, have negative impact on the functional lifespan of contemporary viable spermatozoa which consequently result in irreversible dysfunction that reduces their fertility potential, and ultimately results to death [5]. In addition, the weakened subpopulation in the frozen-thawed semen may die during semen handling while performing assisted reproduction technology (ART) which will add to the existing harmful stresses to the accompanying viable sperm population. It is relevant, therefore, to minimize the toxic effects of dead, senescent, and abnormal spermatozoa by their removal from the semen sample. Previous studies identified ubiquitin-activating enzyme (UBA1) as an enzyme required for sperm capacitation, acrosomal exocytosis and sperm-egg coat penetration during porcine fertilization [6] and this was followed by an attempt to discard subfertile spem populations by using a dual ubiquitin-PNA sperm quality assay with ubiquitin as a surface marker of defective bull sperm, and PNA-lectin, a surface marker of damaged bull sperm acrosomes, for flow cytometric semen evaluation [7]. Recently, studies on nanotechnology-based approach of enrichment of semen with best spermatozoa by targeting damaged boar sperm and enrichment of semen doses with highly motile, viable, and fertile spermatozoa have been reported [8, 9]. Although the above reports present their own benefits and contributions, there are still some constraints associated with such techniques such as cytotoxicity and requirement for analytical procedure such as flow cytometry which may pose insult to the sperm. Therefore, a facile and non-invasive sperm separation technique that reduces clinician skill requirements for sperm purification process is desirable. Such sperm selection technique prior to cryopreservation; one which can be integrated into the cryopreservation protocol that will affect outcomes in terms of fertilization and pregnancy rates is expected to find wide application in animal reproduction. Our approach based on thermotactic forces and fluid flow satisfies the above requirements.

The goal of cryopreservation is to preserve maximum number of post-thawed viable normal spermatozoa and maintain the original pre-freezing sperm quality parameters including structural integrity, viability, motility, DNA integrity, and biological function related to fertilization competence. However, despite the application of sperm cryopreservation as a routine procedure for AI, approximately 50% of spermatozoa do not survive the freezing-thawing process [10]. The process of cryopreservation impairs sperm function and fertility. Spermatozoa suffer from cold shock during cryopreservation [10] and this results in lower post-thaw sperm survival, and for those that survive but cryocapacitated, a reduced longevity in the female reproductive tract [11]. The negative effects of cryopreservation on the sperm includes in addition to cold shock, other damages such as osmotic stress, and alterations in membrane fluidity and permeability [12]. Cryopreservation protocols for bull semen to be used for AI in the dairy industry have been developed in the 1950s [13] and yet advances in cryopreservation techniques have progressed slowly. Moreover, bovine fertility is thought to have declined over the last few decades. An attempt to increase the cryosurvival of bull sperm by the addition of cholesterol to the membrane of bull sperm prior to cryopreservation has been reported to yield a modest 60% motility and 55% viability, and which has not translated into an increased *in vivo* fertility [14, 15].

Our study presents an approach to reduce sperm damage and improve sperm cryosurvival, and consequently achieve high pregnancy rate by semen processing prior to cryopreservation. We attempted to improve fertility of the cryopreserved sperm by devising a new approach to semen processing in order to mitigate the detrimental effects of cryopreservation on sperm function. A sperm selection procedure based on the combined mechanism of rheotaxis and thermotaxis (SSRT) of high quality spermatozoa confined in a slow rotating flow of non-Newtonian fluid was employed to eliminate the dead, damaged, immotile, or dying spermatozoa. Selection of a distinctive population of motile and viable spermatozoa with intact membranes before subjecting it to the cryopreservation process resulted in a compelling elevation of pregnancy rate of cows.

Our preceding study demonstrated that selection of high quality spermatozoa by microfluidic selection of post-thawed semen by rheotaxis could select superior bovine sperm subpopulations, and subsequent fertility was assessed and validated *in vivo* by artificial insemination of cows [4]. The selection of high quality spermatozoa prior to cryopreservation is desirable. However, the major downside of microfluidic sperm selection technique is the limitation in sperm volume that could be processed. The technique is best designed for *in vitro* fertilization (IVF) using frozen-thawed semen and is not practical for large volumes such as raw semen. Nonetheless, we recognized the beneficial effects of freezing selected bovine sperm samples to obtain higher post-thaw quality, and the potential to improve pregnancy rate. This prompted us to develop the current platform of semen processing system based on rheotactic and thermotactic fluid sorting of motile and functional spermatozoa confined in a rotating flow under carefully defined and controlled macroscale conditions to minimize cryoinjury and maximize recovery of viable cells for AI in livestock production. The prefreeze processed cryopreserved semen in straw can be utilized directly for AI without further processing. The utility of the new straw containing the semen subjected to pre-freeze sperm sorting can also be potentially extended beyond AI applications such as IVF.

## Materials and methods

### Reagents

All chemicals were reagent grade and were purchased from Sigma-Aldrich Japan (Tokyo, Japan), except for SP-TL buffer which was purchased from Caisson Laboratories, Inc. (Smithfield, UT).

### Japanese Black bovine bull semen collection

Bovine bull (*Bos taurus*) ejaculates from Two Japanese Black AI bulls at Saga Prefectural.

Livestock Experiment Station, Japan with known fertility were used in this study. Semen collection was performed using a dummy cow and artificial vagina. The first and second ejaculates were collected separately at 30–40 min intervals and mixed together for SSRT method and cryopreservation process. Sperm motility was examined under light microscopy, and subjectively classified into the 5 grades (+++: progressively motile at a high speed, ++: progressively motile at a moderate speed, +: motile at a low speed, ±: motile without progression −: immotile). The proportions of sperm with +++ and ++ grades were defined as motile sperm. These evaluations were performed independently by four practitioners and the mean value was used as the value for motile sperm. Currently, bull pre-breeding examination in Japan includes visual microscopy of ejaculates. Although CASA provides objective measurements in laboratories, it is rarely used on-farm. Our evaluations include both visual assessment by mass sperm motility (motility mass score, MMS) using a light microscope with heated stage and CASA. The semen qualities by MMS evaluated by four technicians, and the total motility by CASA were described in Table 1. The concentration of ejaculates were 14.4 billion/mL and 17.6 billion/mL from Bull#1 and Bull#2, and pH of 6.7 and 6.6, respectively. All ejaculates had a milky-white color. Mass motility scoring of semen sample obtained by a flame-sterilized platinum wire loop was measured by viewing under a microscope with heated stage and at low magnification (20-fold).

**Table 1:** Evaluation of semen quality based on MMS score by quantitative microscopic observation and total motility (%) by CASA

Sire			Prefreeze processed by SSRT	Control straw
Bull #1	Cooled at 4 °C	MS score total motility	70% +++ 87% (221/255)	60% +++ 86% (278/324)
	Frozen-thawed	MS score total motility	50% ++ 86% (278/328)	50% ++ 77% (125/163)
Bull #2	Cooled at 4 °C	MS score total motility	80% +++ 93% (339/363)	75% +++ 84% (125/148)
	Frozen-thawed	MS score total motility	60% ++ 79% (326/412)	45% ++ 68% (116/171)

MS score for fresh semen of Bull #1 = 90% +++; MS score for fresh semen of Bull #2 = 90% +++

### Sperm selection based on rheotaxis and thermotaxis (SSRT) platform

**Fig. 1:**
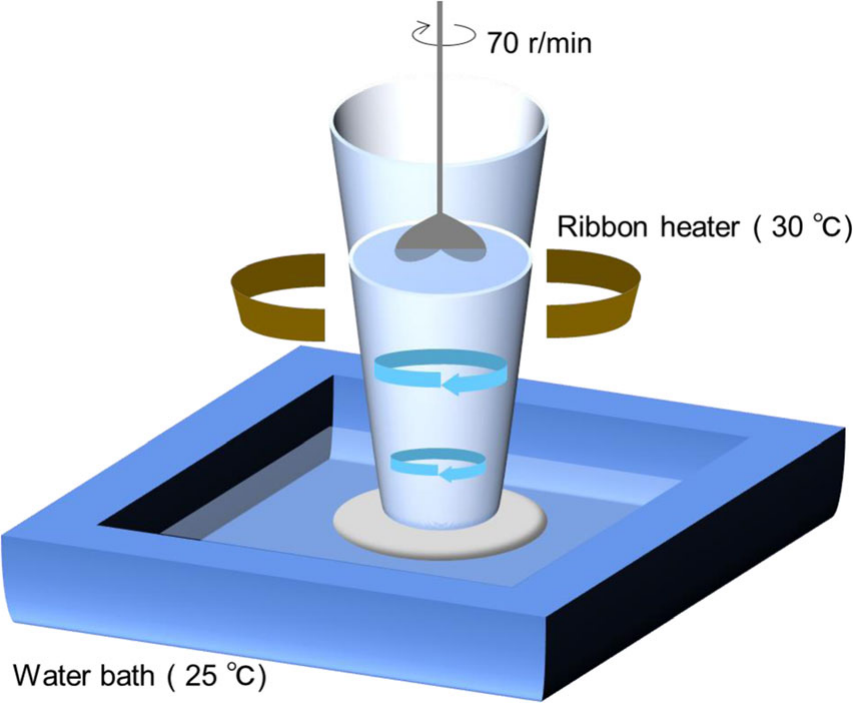
Schematic representation of the prefreeze semen processing technique based on sperm selection by rheotaxis and thermotaxis (SSRT) method. The platform includes a controlled condition of slow and uniform rotational flow (70 r/min) of Newtonian fluid and temperature gradient (25 °C to 30 °C) for 20 min

This study introduced the SSRT platform described in Fig. 1 that utilized an apparatus for prefreeze semen processing. It consists of a conical cylinder that is made of glass and a rotating mixer (Front Lab FLOS20-S) attached with a custom-made rotating two-blade paddle of closely controlled dimension and shape, which is made of acrylonitrile butadiene (ABS), a thermoplastic polymer. This paddle which is connected to a motor generates the force that drive fluid motion and creates a slow circular fluid motion maintained at 70 rpm during the prefreeze processing for 20 min. The ejaculated semen used in the prefreeze processing was extended with the first diluent composed of 113.63 g/L of 2-amino-2-hydroxymethyl-1-3-propanediol [*tris* (hydroxymethyl) aminomethane, 6.96 g/L of citric acid, 3.75 g/L of *D-* (−) fructose, 15.9 g/L of lactose monohydrate, 27 g/L of *D*- (+) raffinose pentahydrate, 600,000 units of penicillin, 600 mg of streptomycin, minus the egg yolk. The strategy was very simple using the principle of rheotaxis and thermotaxis of mammalian spermatozoa. The extended fresh semen was contained in a conical glass vessel which was attached with a heater that was connected to a temperature controller SBX-303 (Sakaguchi TTM-004). The slow but uniform flow characterized as laminar flow and uniquely defined by the Reynolds number, Re < 1, was initiated and maintained by the rotating paddle placed at the center of the conical vessel on top. It is assumed that the ascent of the shear stress led to the increase in the sperm velocity with the direction of the sperm velocity opposite to that of the rotating flow. The temperature gradient was provided at 25 °C at the bottom layer and 30 °C at the upper layer. This processing lasted for 20 min afterwhich the upper layer (70% of total volume) was harvested and immediately added with first diluent plus egg yolk (20%) then slowly cooled to 4 °C inside a constant temperature chamber for treating semen (D15 Fujihira Industry Co., Ltd., Japan). Samples were then slowly added with second diluent composed of egg-yolk-Tris-glycerol extender as 2 equal volumes according to the usual industry procedures. The final glycerol concentration was 7% and for egg yolk was 15%. Extended sperm were then packed into 0.5 mL polyvinylchloride straws using straw filling and sealing machine (Straw Machine T-10, Fujihira Industry Co., Ltd). The final concentration was 3 × 10^7^ sperm/straw. Freezing was done using a programable freezer (Mini-DigiCool 1400, IMV Technologies, France). The straws were then directly plunged in liquid nitrogen. For sperm quality analyses semen in straws were thawed in a water bath at 37 °C for 30 s. Additional file 1 shows the comparison between cryopreservation process of conventional and SSRT subjected straws.

### Post-thaw evaluation of viability, motility, mitochondrial activity, DNA fragmentation and capacitation status

Evaluation of sperm viability was performed by staining the spermatozoa using the Live/Dead.

Sperm Viability Kit (Thermo Fischer Sci Inc., Molecular Probes, Eugene, OR, USA) as per the manufacturer’s guidelines. The post-thawed samples of prefreeze-SSRT selected spermatozoa and unprocessed control were added with SYBR 14 (final concentration 100 nmol/L), vortexed and incubated for 10 min at 37 °C, followed by the addition of propidium iodide (final concentration 12 μL) then 5 min incubation at 37 °C. Aliquots were placed on a prewarmed slide and each aliquot was covered with a coverslip and assessed under Nikon Ti-U fluorescence microscope.

The total motility of post-thaw sperm was assessed by computer-assisted sperm analysis (CASA). Cryosurvival rate (%CSR) based on motility was calculated using the formula: 100 × post-thaw total motile sperm/pre-freeze total motile sperm.

The effect of prefreeze SSRT method on the motility index of bovine spermatozoa at different post-thaw incubation time was assessed by computing for the motility index data which was derived from multiplication of VAP (Average Path Velocity) over BCF (Beat Cross Frequency). The results were compared with that of the unprocessed control.

To evaluate the mitochondrial activity based on the mitochondria membrane potential, aliquots from the post-thawed samples of the cryopreserved SSRT straw and unsorted control straw were stained by the lipophilic cationic probe 5,5′,6,6′-tetrachloro-1,1′,3,3′-tetrathylbenzimidazolecarbocyanine (JC-1) (MitoProbe JC-1 assay kit; Invitrogen) which differentially label mitochondria with high (orange color of multimeric or J-aggregates) and low membrane potentials (green color for monomeric form) [16]. Subsequent analysis was performed by placing samples under a coverslip and observing with Olympus BX51 fluorescence microscope.

For evaluation of chromatin integrity by measurement of DNA fragmentation, the terminal deoxynucleotidyl transferase-mediated deoxyuridine triphosphate-nick-end labelling (TUNEL) assay was employed to assess the nicked DNA in samples from pre-freeze SSRT processed semen and unprocessed control by following the manufacturer’s instruction (In Situ Cell Death Detection Kit, Fluorescein, Roche, Indianapolis, IN, USA) and as published [16]. The method was presented in detail in our previous paper [4]. The DNA fragmentation index determined by the TUNEL assay [16] referred to as sperm TUNEL index was expressed as a percentage of the total population.

The capacitation status of viable bovine spermatozoa was assessed using the dual staining method by following the procedure described by Fraser et al. [17] with some modifications. The chlortetracycline (CTC) fluorescent technique detects changes in the plasma membrane of the bovine spermatozoon [17]. Dual staining of spermatozoa with the supravital fluorescent dye (Hoechst 33258) allows for determination of cell viability prior to CTC analysis therefore, avoiding the assessment of dead spermatozoa as acrosome reacted cell [18]. Spermatozoa with CTC fluorescence uniformly over the entire head (F pattern) were classified as intact and non-capacitated while capacitated spermatozoa were characterized by a fluorescence-free band on the post acrosomal region, and acrosome reacted spermatozoa (AR) showed a nonfluorescent head or a thin fluorescent band on the equatorial segment.

### Field fertility trials by AI of nulliparous heifers and repeat breeders

To evaluate the effect of semen manipulation and selection based on the SSRT method prior to cryopreservation, straws of SSRT selected sperm were cryopreserved and used in artificial insemination (AI). A total of 12 animals were employed consisting of 4 nulliparous Japanese Black and 8 nulliparous Holstein repeat breeders. A cow is called a repeat breeder when it has failed to conceive from three or more regularly spaced services in the absence of detectable abnormalities. The causes of repeat breeding are fertilization failure and embryonic mortality. The fertility of prefreeze SSRT treated sperm was assessed by a sample size of 12 cows. This field test of small sample size is considered indispensable prior to conduct of any large-scale application.

The cows (4 Japanese Black heifers and 8 Holstein repeat breeders) were observed for natural estrus and inseminated under the am-pm rule. AI was performed using transcervical intrauterine technique by different technicians (*n* = 3) to eliminate technician bias. AI field experiments were conducted from early to late summer. Pregnancy verification was performed by transrectal ultrasonography after 30 and 50 days of insemination.

## Results

### Assessment of post-thaw sperm recovery and viability

**Fig. 2:**
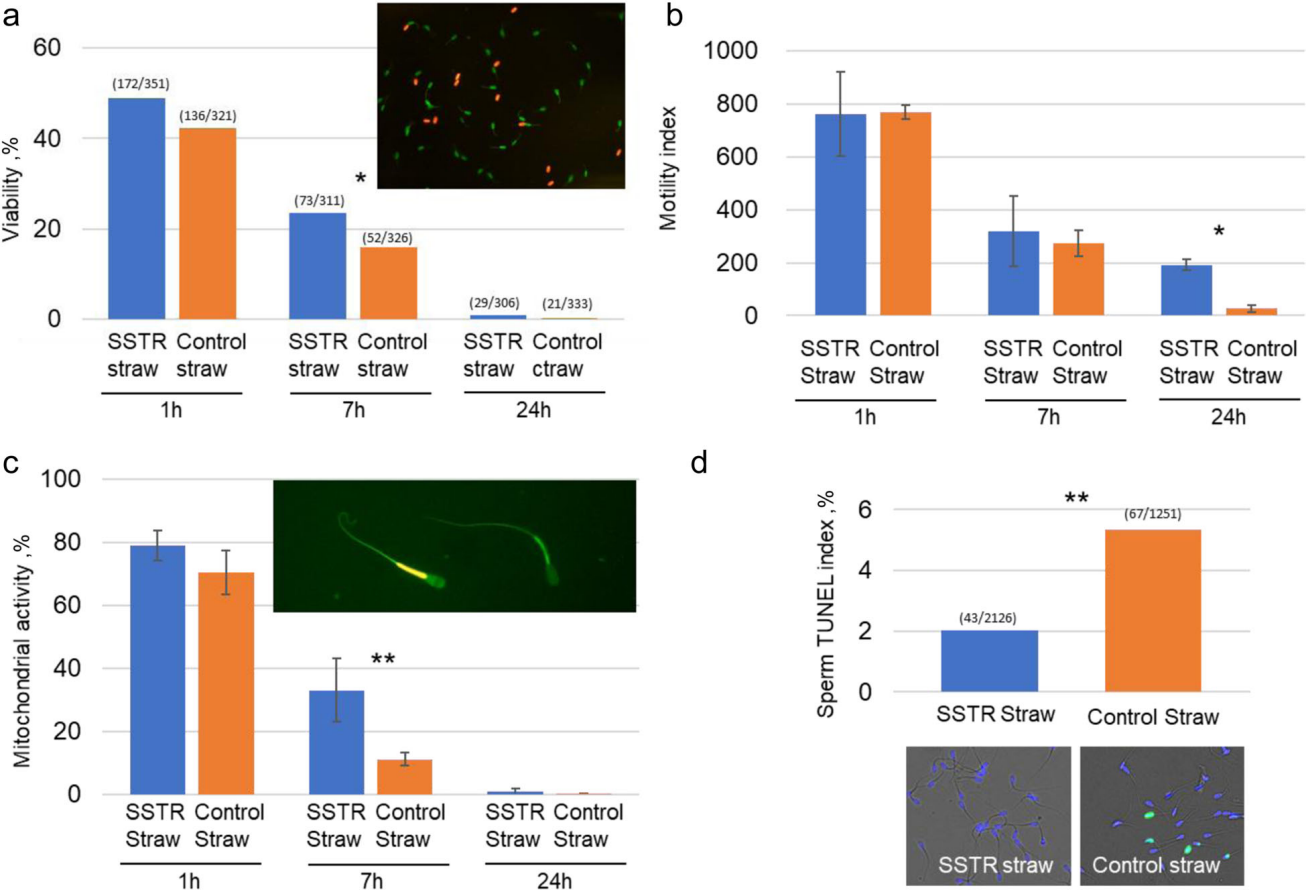
**a** Effect of semen processing by SSRT method prior to cryopreservation on post thaw sperm viability in bovine spermatozoa, and representative images of spermatozoa in dead (red) and live state (green). Viability values between SSTR and control straws differ significantly at *P* < 0.05 (Chi-squared test) for 7 h incubation. **b** Effect of semen processing by SSRT method prior to cryopreservation on the motility index (VAP × BCF, measured by CASA) of bovine spermatozoa at different incubation time (1 h, 7 h, 24 h). Viability values between SSTR and control straws differ significantly at *P* < 0.05 (*t*-test) for 24 h incubation (**c**) Effect of semen processing by SSRT method prior to cryopreservation on post thaw mitochondrial activity of bovine spermatozoa, and fluorescence photomicrography of sorted spermatozoa stained with JC-1 probe showing orange fluorescence of the sperm midpiece indicating hMMP and green fluorescence indicating low mitochondrial membrane potential. Values between the SSTR and control straws at 1 h and 24 h are not significantly different. Values at 7 h are significantly different at *P* < 0.01 (*t*-test). **d** Effect of semen processing by SSRT method prior to cryopreservation on post thaw DNA integrity measured by TUNEL index (%) that indicates DNA fragmentation. Each spermatozoon was assigned to contain either normal (blue nuclear fluorescence due to Hoechst 33342) or fragmented DNA (green nuclear fluorescence). There are 4 straws analyzed for each treatment (*n* = 4 per treatment), and every straw was subjected to 4 times CASA measurements, mitochondrial activity, and TUNEL assay. Values differ significantly at *P* < 0.01 (Chi-squared test)

High quality spermatozoa were selected by processing ejaculated bull semen based on rheotactic and thermotactic fluid sorting of motile and functional spermatozoa confined in a rotating flow under carefully defined and controlled macroscale conditions as shown in Fig.1. This semen processing technique resulted in the improvement of cryosurvival by removing dead, damaged and dying spermatozoa before freezing. There were no observed detrimental effects to bull spermatozoa after their exposure to slow uniform rotating fluid flow and temperature gradient as demonstrated by results below (Fig. 2). Selected spermatozoa showed improved cryosurvival compared to unselected control, and survived longer after thawing (Fig. 2a).

### Evaluation of semen quality by mass sperm motility (motility mass score) and CASA system

Traditional assessment of post-thaw sperm viability and motility by visual measurement based on gross motility (or mass activity) was employed. Gross motility, or the amount of swirling (or wave motion) present in an undiluted semen sample, gives the motility mass score (MMS) and is a function of both sperm concentration and individual motility. This technique is routinely used in livestock breeding centers in Japan and based on the assessment would determine whether to proceed with the cryopreservation or not. The MMS is expressed as the percentage of motile (viable) cells and motility. The percentage of motile cells (%) is estimated by visual measurement, with the number of spermatozoa in all viewing fields taken as 100. The motility is classified as follows: +++ vigorous movement or the greatest forward movement that looks like a vortex or swirling motion, ++ active forward movement, + weak movement. The MMS was assessed by 4 technicians who have sufficient experience in bull semen evaluation and cryopreservation. The effect of sperm preparation on post-cryopreserved sperm was evaluated by comparison of mass sperm motility before and after cryopreservation (Table 1). Evidently, based on MMS, sperm sorting prior to cryopreservation enhanced sperm cryosurvival. Table 1 shows that the prefreeze-processed spermatozoa by SSRT method have higher MMS after cooling to 4 °C than the control group for indicating that tolerance to cooling was enhanced by prefreeze sperm selection compared with the control groups. Freezing induced a 20% decrease in the MMS of SSRT subjected straw for both bulls. The control groups demonstrated a 10% decrease in MMS (Bull #1) and a 30% decrease (Bull #2), suggesting individual bull variation in terms of freezability. It is worthy to note that an improvement in cryopreservation was achieved by prefreeze processing using the SSRT method as evidenced by the enhancement of post-thaw MMS of Bull #2 (45%++ control vs. 60%++ prefreeze processed). In addition, variation in cryopreservation tolerance of the two bulls was observed as depicted by the prefreeze processed and control straws. Cryopreservation reduces survival of spermatozoa to a great extent but this was mitigated by sperm processing using the SSRT method prior to cryopreservation.

Sperm concentration was determined by using cell counter as well as hemocytometer. Moreover, computer-assisted sperm analysis (CASA) system was used for sperm motility analysis.

### Viability

The viability of cryopreserved bovine spermatozoa from straws containing pre-freeze processed semen by SSRT and the control is presented in Fig. 2a. Significant differences were observed between the two straws in terms of mean percentage of sperm viability at different incubation periods. At 1 h, 7 h, and 24 h incubation time, spermatozoa subjected to prefreeze SSRT showed higher viability than the control however, significant difference was observed particularly at 7 h (*P* < 0.05). A longer *in vitro* life span was achieved by prefreeze processed spermatozoa than the control.

### Cryosurvival rate (%CSR) based on motility

The % CSR was calculated using the formula: 100 × post-thaw total motile sperm/pre-freeze total motile sperm. The semen subjected to sperm selection processing prior to cryopreservation had significantly higher cryosurvival rate compared to control (98.6 ± 1.04 vs. 88.1 ± 3.4, *P* < 0.01 for Bull #1; and 88.5 ± 3.7 vs. 79.4 ± 1.51, *P* < 0.01 for Bull # 2 as presented in Table 2. Subsequent to thawing of cryopreserved bovine bull semen, the CSR of semen subjected to prefreeze processing by SSRT method increased by 9% to 10% as compared to non-processed control thus demonstrating the positive effect of prefreeze SSRT method by increasing the survival rate after cryopreservation.

**Table 2:** Cryopreservation rates (% CSR) of semen subjected to sperm selection process prior to cryopreservation

Sire	Prefreeze processed by SSRT (% CSR)	Control (% CSR)
Bull # 1	98.6 ± 1.04	88.1 ± 3.03
Bull # 2	88.5 ± 3.70	79.4 ± 1.51

Values are significantly different at *P* < 0.01 (Bull#1) and *P* < 0.05 (Bull#2) (t-test)

### Assessment of the effect of prefreeze SSRT method on the motility index of bovine spermatozoa at different post-thaw incubation time

Assessment of sperm motility has been shown as one of the important factors to predict the functional capacity of bovine sperm cells. The motility index data derived from multiplication of VAP over BCF was used to evaluate the effect of prefreeze SSRT method. Although post-thaw motility index was higher at 1 h incubation for the control straw, considerable reduction was observed at 7 h incubation period, and such reduction was greater in comparison with the control straw (Fig. 2b). At 24 h incubation, the control showed a 98% decrease in motility index while 84.5% reduction was observed in the SSRT method subjected semen straw. Motility index was higher for the prefreeze processed semen than the control suggesting an enhancement in cryosurvival based on motility and improvement of semen quality by motility characteristics.

### Evaluation of the effect of prefreeze SSRT method on the post thaw mitochondrial activity

The assessment of mitochondrial membrane potential (MMP) by fluorophore JC-1 revealed no difference in MMP at 1 h post-thaw incubation (Fig. 2c). However, the effect of *in vitro* incubation time on the survival function of mitochondrial activity was observed at 7 h incubation. Prefreeze SSRT processed semen showed a significantly higher percentage of MMP than the control (*P* < 0.01). The reduction in the survival function of mitochondrial activity in prefreeze processed semen after exposure time of 7 h (56%) was lower compared with the unprocessed semen (86%). A gradual cessation of mitochondrial activity within the 24 h period was evident in prefreeze processed semen in contrast to the drastic decline in unprocessed control. This reflects that dysfunctional mitochondria were present more in the control group which can be due to the presence of non-viable, damaged and senescent spermatozoa than the prefreeze processed semen and the disparity in the alteration of mitochondrial activity became more evident with incubation time. Selection of high quality and motile sperm had a positive effect as indicated by higher mitochondrial activity in prefreeze sorted compared with the control (Fig. 2d). While time course of the decline of sperm mitochondrial activity was significantly slower in prefreeze-sorted spermatozoa, contrastingly, the control demonstrated a rapid impairment in mitochondrial activity. These results indicate the positive effect of macroscale rheotactic and thermotactic sorting on prolongation of sperm mitochondrial activity life span and maintenance of high sperm quality as assessed by the mitochondrial functionality.

### Effect of prefreeze SSRT method on the post thaw DNA integrity

DNA integrity is one of the parameters of sperm quality associated with fertility hence, DNA fragmentation (DFI, %) of post-thawed spermatozoa was evaluated by using the terminal deoxynucleotide transferase-mediated dUTP nick end labelling (TUNEL). The post-thawed DFI of prefreeze SSRT selected spermatozoa was significantly lower than the unprocessed control (2% vs. 5.5%, *P* < 0.01, Fig. 2d). Such reduction in DNA fragmentation achieved by sperm processing by SSRT before cryopreservation allowed for effective recovery of not only viable and motile but functional and high quality spermatozoa as demonstrated by high DNA integrity. This data supports the significantly higher pregnancy rate of cows inseminated with prefreeze SSRT selected spermatozoa compared with cows inseminated with the conventionally prepared semen (control).

### Influence of sperm selection prior to cryopreservation by SSRT method on pregnancy rate of artificially inseminated cattle

The fertility trials (AI) that have been carried out indicate that the selected spermatozoa prior to cryopreservation may have enhanced fertilizing capacity compared to unselected control (Table 3). During AI, we used the routine cervical insemination technique during natural estrus (am-pm rule) to inseminate the 4 Japanese Black heifer and 8 repeat breeder Holstein cows with post-thawed semen from 2 bulls which were ejaculated, subjected to SSRT method, and cryopreserved on the same day. The AI using straws containing semen processed before cryopreservation was performed in early, mid and late summer of 2017 and 2018 (late of May to late September) in Japan. The pregnancy rates of the bull semen processed by SSRT method prior to freezing is quite impressive (Bull #1 = 100%; Bull #2 = 83.3%) and has far exceeded the unprocessed control (Bull #1 = 44%; Bull #2 = 59%). These results present an evidence of improvement in cryosurvival and quality of spermatozoa by prefreeze sperm selection which allow sperm cells a maximum potential for fertilization. The pregnancy outcome among repeat breeders revealed an impressively rate of 87.5% (7 out of 8 repeat breeders). Repeat breeder syndrome is a frustrating problem affecting reproductive management of dairy cows. This encouraging result obtained from AI of repeat breeders which were in spontaneous estrus suggest the promising potential of SSRT strategy in the management of repeat breeders by improving the conception rate that will consequently reduce the culling rate. We attribute the increased pregnancy outcome to SSRT method which selected high quality sperm with improved sperm parameters and increased fertilization potential.

**Table 3:** Clinical data on pregnancy rate of cows inseminated with semen straw containing sperm cells selected prior to cryopreservation

Cow		History of failed reproduction	Sire	Pregnancy
Heifer	Japanese Black #1	None	Bull #1	Yes
	Japanese Black #2	None	Bull #2	Yes
	Japanese Black #3	None	Bull #1	Yes
	Japanese Black #4	None	Bull #2	Yes
Repeat breeder	Holstein #1	2 × ET, 2 × AI	Bull #1	Yes
	Holstein #2	2 × ET, 3 × AI	Bull #2	Yes
	Holstein #3	2 × ET, 2 × AI	Bull #1	Yes
	Holstein #4	2 × AI	Bull #2	No
	Holstein #4	3 × AI	Bull #1	Yes
	Holstein #5	2 × ET, 3 × AI	Bull #1	Yes
	Holstein #6	4 × AI	Bull #1	Yes
	Holstein #7	2 × ET, 2 × AI	Bull #2	Yes
	Holstein #8	4 × AI	Bull #2	Yes

*ET* embryo transfer, *AI* artificial insemination using conventional frozen semen

AI for Holstein #4 had been performed twice; first AI resulted in nonpregnancy, second AI achieved pregnancy

Pregnancy rates of Japanese Black and Holstein cows (*n* = 12) inseminated with pre-freeze selected sperm cells: Bull #1 = 100%, *n* = 7; Bull #2 = 83.3%, *n* = 6, Total pregnancy rate = 92.3%

Pregnancy rates of control Japanese Black cows inseminated with conventionally prepared semen from the 2 sires; Bull #1 = 59.2%, *n* = 76; Bull #2 = 44.4%, *n* = 36, Total pregnancy rate = 54.5%

Values between SSTR and conventional straws differ significantly at *P* < 0.01 (Chi-squared test)

### Influence of prefreeze SSRT method on post thaw acrosome reactivity

**Fig. 3:**
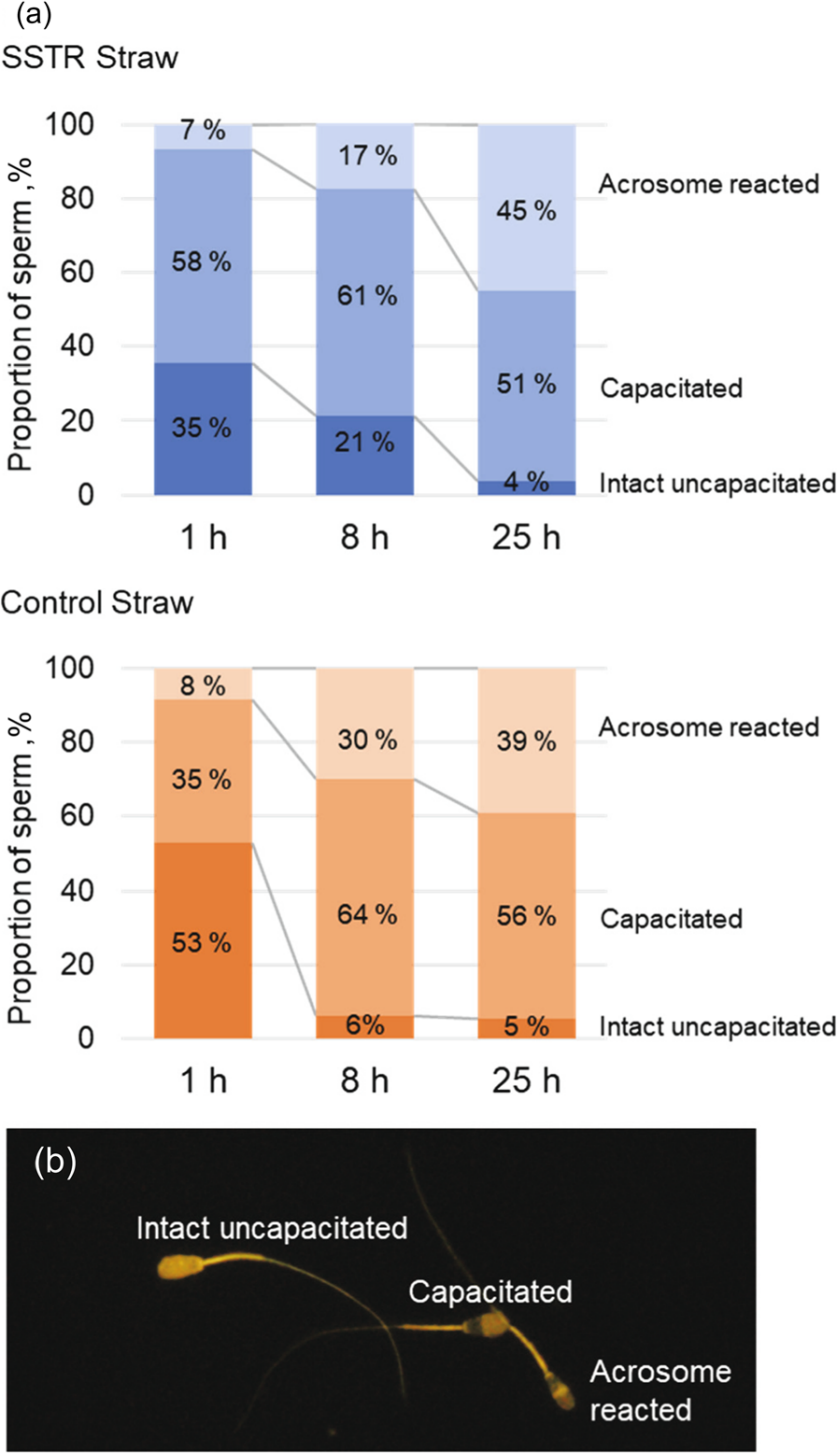
**a** Effect of semen processing by SSRT method prior to cryopreservation on post thaw acrosome reactivity. The proportion of capacitation and acrosome-reacted, and uncapacitated spermatozoa were assessed by CTC assay. Each sample was incubated at 1 h, 8 h, and 25 h. Replicates of 2 for 192–297 sperm per replicate of SSRT and control sample per replicate. **b** Fluorescence photomicrography of sperms stained with CTC probe

The membrane status of post-thawed spermatozoa from prefreeze processed and unprocessed control samples was evaluated and compared. The proportion of intact (uncapacitated), capacitated, and acrosome-reacted as evaluated by CTC assay revealed that there are intrinsic differences in the proportion of the acrosome reacted (AR) over time as observed in the prefreeze processed and unprocessed control (Fig. 3), characterized by an increasing trend. It is noteworthy to consider that prior to CTC staining, the post-thawed semen was washed and resuspended in a capacitating medium; modified SP-TALP. At 8 h incubation, the control group yielded more AR than the prefreeze processed group while the proportion was almost the same at 24 h incubation. The proportion of capacitated spermatozoa was a little higher for the prefreeze processed than the control at 1 h incubation. However, at 8 h, considerable increase in the percent of capacitated sperm was observed in the control while the capacitated proportion in the prefreeze processed leveled off and slightly decreased at 24 h. The major difference on the membrane status between the prefreeze processed and control was evident on the proportion of intact and uncapacitated spermatozoa showing more than thrice as many uncapacitated spermatozoa at 8 h than the control. Such results suggest that processing prior to cryopreservation selected spermatozoa that are capable of capacitation but undergo slow AR than the unprocessed control. Moreover, prefreeze semen processing yielded an increased proportion of uncapacitated sperm at 8 h in contrast to the decreased uncapacitated sperm of unprocessed control.

## Discussion

Animal ejaculates contain non-viable or dead spermatozoa along with damaged and moribund spermatozoa which substantially increase in number when semen is subjected to cryopreservation and thawing that increase risks of damage to these cells. It has been reported that large numbers of non-viable sperm cells has negative influence on the functional lifespan of contemporary viable sperm which consequently result in irreversible dysfunction thus, reducing the fertility potential of the semen [5]. The ejaculates also contain other cells such as leukocytes which may have contributed to radical oxygen species (ROS) generation [19]. Our study aims to highlight the relevance of selecting motile and high quality spermatozoa prior to cryopreservation, assuming that sorting plays relevant effects on the functional lifespan and fertilizing potential of these sorted gametes. Semen processing by rheotactic and thermotactic mechanism prior to cryopreservation was considered as a means to eliminate the damaged and non-viable sperm which has negative influence on the functional lifespan of contemporary viable sperm that eventually cause irreversible dysfunction that consequently reduces their fertility potential and ability to develop healthy embryos. Considering that the number of dead sperm increases in semen over time, and that the presence of dead sperm in the ejaculate to be stored should be considered within the set of harmful stresses posed to the accompanying viable sperm population, we developed a facile but efficient method of motile sperm separation by exposure to slow uniform rotating fluid flow (rhetotaxis) and temperature gradient (thermotaxis) in a macroscale platform. It should also be considered that the motility of sperm is critical to a successful fertilization. Spermatozoa are extremely efficient at swimming against a current just like salmon traveling upstream to spawn [20, 21]. In contrast with dead sperm which resembles passive particles [22], motile sperm exhibit rheotaxis [23–25]. Our previous study presented evidence of spermatozoa achieving forward propulsion through fluid in microchanels. Considering this principle but in a macroscale, we created the right velocity of a rotating fluid to elicit upward movement of motile and presumably high quality spermatozoa. The fluid which is composed of semen diluted in Fraction A extender without egg yolk (osmolality = 300 mOsm) is classified as a Newtonian fluid with low viscosity. The flow condition is uniquely defined by the Reynolds number, Re < 1. In synergy with this system is the response of sperm cells to temperature gradient which can also elicit upstream motility of sperm.Under the basic sperm medium, sperm motility has been classified into two types: one type of motion by rolling of the body and another type, by 7 planar beating of the flagellum [26–28]. The extender medium used in the SSRT method can be classified as basic medium and considering that bull spermatozoa was subjected to slowly rotating bulk fluid condition, they were classified as bulk swimmers [27], repeatedly rotating their body around its axis that is basically due to propagation of a 3D helical wave along the flagellum [29]. The ascent of shear stress by the uniform flow patterns of the rotating fluid could lead to the increase in the sperm velocity with the direction of the sperm velocity opposite to that of the flow. The fluid flow condition in the SSRT method can be explained by the dynamic “Similitude concept” wherein geometric similarity and kinematic similarity are observed. Another physical environment that worked on to facilitate sperm migration to the upper layer of the conical vessel is thermotaxis It has been reported that the migration of sperm in the female reproductive tract associated with fertilization is regulated by thermotaxis [30, 31]. The temperature gradient allows sperm to orientate their swim away from the utero-tubal junction (low temperature) towards the warmer ampulla where the oocyte awaits [32–34]. Thermotaxis of bull sperm was confirmed involving both calcium channels and intracellular stored calcium [34]. Our SSRT method utilized this combined mechanisms of migration dynamics sensitive to temperature and rheological stimuli, and the obtained data indicate not only improvement of cryosurvival of prefreeze selected spermatozoa but compelling results in AI provide a solid evidence that pre-selection of high quality spermatozoa prior to cryopreservation by SSRT method is a robust means of improving the fertilization potential of bull semen.

In cattle, bull semen varies widely in terms of freezability outcome. Our SSRT method prior to freezing has been shown to reduce such variation by improving the cryosurvival of spermatozoa via elimination of dead, damaged and dying sperm cells in the ejaculate and their deleterious effects. Such improvement was particularly demonstrated by Bull #2 wherein cryosurvival of 45% (failed to pass for commercial market) was improved to 60% (passed for commercial market). Such data was supported by the higher % CSR of prefreeze-SSRT selected spermatozoa (89%) compared with the control (52%) (Table 2). Moreover, it should be considered that AI was conducted during the summer season when cows were subjected to heat stress. The elimination of bull variability and AI technician variability demonstrated the robustness of the new straw prepared by this strategy. The increased pregnancy rate in repeat breeder cows inseminated with SSRT selected sperm suggests that SSRT method can serve as an excellent tool for improvement of pregnancy rate not only in normal heifers but also repeat breeders. The result also provides insight and opportunities as to the possible role of high quality sperm selection in management of repeat breeding. SSRT is a promising technique that needs large fertility trials.

Sperm motility was evaluated in the analysis of fertilization potential and cryosurvival considering that it is an index of structural integrity and viability of spermatozoa [35]. One of the deleterious effects of cryopreservation is the reduction of sperm motility. Such effect was mitigated by preselection of high quality and motile spermatozoa before cryopreservation. In addition, a longer life span was achieved by prefreeze-SSRT subjected spermatozoa. It has been reported that non-viable spermatozoa due to programmed death or apoptosis, more likely prior to ejaculation, and necrosis, resulting from severe cell stress due to an external source, have negative impact on the functional lifespan of contemporary viable spermatozoa which consequently result in irreversible dysfunction that reduces their fertility potential, and ultimately results to death [36]. Moreover, the weakened subpopulation in the frozen-thawed semen may die during semen handling while performing assisted reproduction technology (ART) which will add to the existing harmful stresses to the accompanying viable sperm population. SSRT method had minimized the toxic effects of dead, senescent, and abnormal spermatozoa by their removal from the semen sample prior to freezing. Elimination of dead and damaged sperm will likely extend the lifespan of the surviving spermatozoa in the cow genitalia. A longer sperm life span would mean wider window for timing of sperm capacitation and ovulation, as reflected by the time relationships among reproductive events and fertile lifespan of bovine spermatozoa and oocyte [4]. The functional integrity and timely transport of spermatozoa in the female genital tract is crucial for maximizing the chance of fertilization and successful pregnancy. This might be one of the factors that could explain the high pregnancy rate observed in prefreeze-SSRT subjected semen.

Alteration in mitochondrial activity was slower in SSRT prefreeze processed semen than the control (Fig. 2c). SSRT had a positive effect, as indicated by higher mitochondrial activity in sorted compared with the unsorted control. The time course of the decline of sperm mitochondrial activity was significantly slower in SSRT preselected semen. Contrastingly, the control demonstrated a rapid impairment in mitochondrial activity. These results indicate the positive effect of SSRT prefreeze processing on prolongation of sperm mitochondrial activity life span and maintenance of high sperm quality as assessed by the mitochondrial functionality. Mitochondria play a vital role as bioenergetic sources for spermatozoa vitality, and motility. The main functional role of sperm mitochondria is related to energy production (by ATP) and sperm mitochondrial functional characteristics are closely associated with spermatozoa motility [37–39] and better fertilization potential [40]. The changes in the mitochondrial integrity and functionality, namely defects in mitochondrial structure, mitochondrial genome, as well as disturbances in mitochondrial membrane potential or altered oxygen consumption, have been correlated with loss of sperm functions; sperm motility, in particular [37]. The survival function in terms of mitochondrial activity overtime was higher in the prefreeze processed sperm than the control. There are reports on the positive correlation between total motility and MMP [41–45], and the close relation between integrity of mitochondrial membrane and sperm progressive motility [41]. Because sperm selection was based on motility, it was expected that preselected sperm prior to cryopreservation show a well-maintained motility over time than the unselected sperm. Metabolic energy in the form of ATP acts as a molecular motor to generate force for the flagellar movement. This energy is generated in the mitochondria through oxidative phosphorylation [46] to facilitate efficient propulsion for the sperm to reach the oocyte and to penetrate through zona pellucida or by anaerobic pathway via glycolysis in the cytoplasm [44]. Bull spermatozoa with high MMP are expected to have high fertilizing potential as evidenced by the compelling high pregnancy rate of prefreeze SSRT selected spermatozoa.

DNA integrity is essential for successful fertilization. However, when ejaculates are cryopreserved using routine procedures, dead, damaged, abnormal and moribund spermatozoa are also cryopreserved along with healthy normal sperm that become inevitable source of reactive oxygen species [47]. Cryopreservation also increases the proportion of these detrimental spermatozoa and consequently results in the decrease of DNA integrity of the post-thawed sperm which will concomitantly decrease the fertility and pregnancy rates [48, 49]. It has been reported that cryopreservation process causes DNA damage to mammalian sperm like bull [50]. Spermatozoa carrying nicked DNA may more likely have a negative effect on fertility, with DNA fragmentation being an uncompensable trait. This may be relevant as suggested by the increasing evidence that the integrity of the sperm chromatin at the actual time of fertilization influences embryo survival [48]. DNA-nicked spermatozoa showed adverse effects on early embryonic development [50]. On the other hand, efficient improvement of cryosurvival and post-thaw semen quality through removal of immotile, dead and abnormal spermatozoa by SSRT method was able to exclude these detrimental cells. Spermatozoa carrying nicked DNA may more likely have a negative effect on fertility, with DNA fragmentation being an uncompensable trait. The prefreeze sperm selection by SSRT allowed recovery of spermatozoa with lower DNA fragmentation (higher DNA integrity). The fragmented DNA accumulated in the non-viable and damaged spermatozoa were likely eliminated by the prefreeze selection process in our study. The relevance of improvement of DNA integrity (Fig. 2d) has been supported by the impressive *in vivo* fertility results and livebirths using the prefreeze-SSRT processed semen straws (Table 3). Conversely, poor sperm DNA integrity caused by cryopreservation process may account for some of the infertility in cattle.

The cryopreservation processes also induce precocious capacitation termed “cryo-capaciation” [11] but this does not occur in the entire population as supported by penetration of many oocytes with cryopreserved semen in medium with heparin, a capacitating agent for bull sperm compared with the heparin free [51]. Nonetheless, the cryo-capacitated sperm plasma membrane is fragile and unresistant to spontaneous acrosome reaction and deterioration and finally death in the absence of occurrence of fertilization. Hence, the *in vivo* fertile lifespan of spermatozoa could be restricted particularly with the high proportion of cryocapacitated sperm in the insemination dose. In the experiment, however, it should be noted that the spermatozoa were suspended in a capacitating medium, modified SP-TALP. We assume that the observed capacitation in the prefreeze SSRT-treated spermatozoa at 1 h was true capacitation and not the premature capacitation induced by membrane modification due to cryopreservation. This is supported by the observed slow and controlled capacitation in prefreeze-SSRT-treated sperm in contrast to the unprocessed control. The intact and uncapacitated percentage of sperm was higher in prefreeze-processed than the control at 8 h incubation time. The drastic reduction of proportion uncapacitated sperm in control at 8 h is an evidence that more sperm which were uncapacitated at first but may have partially damaged plasma membrane will demonstrate cryocapacitation following cryopreservation and this become more evident at 8 h while higher proportion of uncapacitated sperm was maintained by prefreeze-processed at 8 h. Moreover, AR was relatively higher in the control than the prefreeze-treated at 8 h indicating that selection of high quality sperm prior to freezing and subjecting the semen under a slow rotating fluid flow condition somehow protects the plasma membrane by probably by a controlled calcium mechanism involved in the onset of capacitation and AR, and another hypothesis is that the environmental fluid condition in the experiment which created a condition that elicits rheotaxis and thermotaxis not only facilitate separation of high quality and motile sperm but ameliorates plasma membrane irreversible damage and controls the onset of capacitation and AR thus increasing the longevity of sperm until the time of meeting the oocyte. It has been reported that cryo-processes involving change in temperature from body temperature to cooling followed by near freezing represents a major stress to the sperm plasma resulting in the rearrangement and destabilization of membrane components, and calcium influx [52, 53]. Some studies suggest that this increased permeability is due to subtle irreversible structural changes to the membrane architecture due to cooling below the thermotropic phase transition temperature of membrane phospholipids. The susceptibility of the sperm to damage by cold shock and cryopreservation results from the lipid-phase transition temperature of the membrane at nonphysiologic temperatures. In bovine sperm, the lipid-phase transition temperature is about 13 °C; the most critical temperature range for cooling sperm is between 15 °C and 5 °C, which determines cold shock, the most sensitive temperature especially for bovine sperm [54]. This can be reduced to 4 °C by an egg yolk-based extender, that’s why in the conventional cryopreservation protocol, semen extender contains egg yolk. Still, lipid components of the bull spermatozoa are significantly reduced due to freezing [55]. There is a general notion that cold resistance is related to membrane fluidity, therefore changes in the fluidity of sperm plasma membranes could increase their cryoresistance. We hypothesize that exposure to synergistic conditions in the SSRT method (fluid flow, temperature gradient, separation of dead and immobile from viable, motile sperm facilitate a preconditioning that may be associated with higher membrane fluidity and consequently cryosurvival. Fresh semen contains cholesterol that acts as a buffer in the phospholipid membranes. Because the phospholipids are pretty flexible (fluid mosaic model) the cholesterol helps to keep them in place. They are placed in between the hydrophobic tails, and therefore it is hydrophobic interactions that decrease the fluidity. However, cryopreservation injury reduces cholesterol and this affecting membrane fluidity. We are tempted to hypothesize that prefreeze processing by SSRT particularly slowly rotating fluid flow also facilitate increase in the cholesterol content of sperm membranes which may be a strategy that can improve sperm quality after freeze-thawing. Considering that functional integrity of the sperm plasma membrane is of primary importance for the cryosurvival and fertilizing abilities of spermatozoa, both of which are successfully achieved in the experiment, we speculate that sperm that accumulated at the 30 °C zone (upper layer) may have increased cholesterol content which subsequently impart cryosurvival property. This preconditioning treatment can have a positive influence on cryotolerance which maybe related to changes in the overall composition of the cell membrane more likely, of cholesterol having a profound effect on the thermodynamic and mechanical properties of lipid bilayers, and influence on the stability and fluidity of sperm [56].

In contrast, in unprocessed control the naturally occurring transfer of cholesterol from bovine sperm to the surrounding medium may be present thus, making the sperm lipid membrane susceptible to cryoinjury. In fact, it has been reported that modification of membrane cholesterol in bull spermatozoa improves post-thaw survival and prevents impairment of sperm function after post-thawing [57]. To elucidate the possible role of SSRT method in providing an environment for stability of plasma membrane and clarify the contribution of cholesterol in protecting the sperm from cryoinjury, we need further investigation and supporting evidences.

## Conclusions

One of the major results in our study is the high improvement in pregnancy rate (93%, *n* = 13) of heifers and repeat breeders. The repeat breeders used in the study were female heifers that failed to get pregnant after three or more attempts. Of the 8 repeat breeders, 7 achieved successful pregnancy and livebirths while all nulliparous heifers inseminated with prefreeze processed semen became pregnant which resulted in livebirths (100% calving rate). It is also important to note that the cows were observed for natural estrus and inseminated under the am-pm rule. The robustness of the strategy is also demonstrated by the fact that the new straw produced form this method erased the technician bias as supported by the different technicians (*n* = 3) that performed the transcervical intrauterine AI technique. The prefreeze SSRT method based on the synergistic effects of slowly rotating fluid flow platform (effect of fluid flow on spermatozoa) and temperature gradient (thermotactic effect on spermatozoa) created an environment that guided motile and viable spermatozoa to self propulsion and migration to the upper layer of the vessel at 30 °C temperature. Therefore, a responsiveness of the sperm to opposite flow and temperature gradient could then be seen as an essential sperm function that can be used at a macroscale to process fresh semen prior to cryopreservation. This strategy attempted to improve fertility of the cryopreserved sperm by reformulating cryopreservation protocols by adding a new but simple approach to enrich semen doses with viable, motile and fertile spermatozoa based on prefreeze SSRT method. This approach results in selected sperm population composed of highly motile and fertile spermatozoa. Our results provide solid evidence that improvement of post-thaw semen quality by motile sperm selection prior to cryopreservation would be beneficial in terms of cryosurvival, and increase in sperm survival and longevity after insemination with frozen-thawed semen in the female reproductive tract thus, optimizing fertilization, embryo development and calving as supported by the favorable results of our field fertility study. Moreover, the SSRT platform eliminates the moribund sperm in the inseminate and improves semen quality and viability, an important contribution to previous works on sperm selection. However, unlike other sperm selection techniques that requires immediate use in AI after the selection procedure, our current method process bull semen and selects motile and fertile sperm prior to cryopreservation; a simple platform integrated to the current cryopreservation procedure used by the AI industry, thus offering a practical but simple way of improving the conception rate. Although our study involves a field test of small sample size, it is considered indispensable prior to conduct of a large-scale application and offers a simple sperm selection technology prior to cryopreservation with a promising means to improve animal conception rate.

## Supplementary information

**Additional file 1.** Detail information of cryopreservation.

## Data Availability

All data generated or analyzed during this study are included in this published article.

## Abbreviations

%CSR: Cryosurvival rate
ABS: Acrylonitrile butadiene
AI: Artificial insemination
AR: Acrosome reacted
ART: Artificial reproductive technologies
ATP: Adenosine triphosphate
BCF: Beat-cross Frequency
CASA: Computer-assisted sperm analysis
CTC: Chlortetracycline
DFI: DNA fragmentation index
IVF: *In vitro* fertilization
JC-1: Mitochondrial Membrane Potential probe (5,5’,6,6’-tetrachloro-1,1’,3,3’-tetrathylbenzimidazolecarbocyanine)
MMS: Motility mass score
Re: Reynolds number
ROS: Radical oxygen species
SP-TALP: Sperm-Tyrode’s Albumin Lactate Pyruvate
SSRT: Sperm selection based on rheotaxis and thermotaxis
TUNEL: Terminal deoxynucleotidyl transferase-mediated deoxyuridine triphosphate-nick-end labelling
VAP: Average Path Velocity

## References

[1] Holt WV (2000). Fundamental aspects of sperm cryobiology: the importance of species and individual differences. Theriogenology.

[2] Alam SS, El Makawy AI, Tohamy AA, Abd Elrahman MM (2015). Effect of seasonal variations on semen quality and fertility of Egyptian water Buffalo (Bubalus bubalis) bulls. Research J Pharm Biol Chem Sci.

[3] Al-Kanaanab A, Königa S, Brügemanna K (2015). Effects of heat stress on semen characteristics of Holstein bulls estimated on a continuous phenotypic and genetic scale. Livestock Sci.

[4] Nagata MPB, Endo K, Ogata K, Yamanaka K, Egashira J, Katafuchi N (2018). Live births from artificial insemination of microfluidic-sorted bovine spermatozoa characterized by trajectories correlated with fertility. Proc Natl Acad Sci U S A.

[5] Roca J, Parrilla I, Gil MA, Cuello C, Martinez EA, Rodriguez-Martinez H (2016). Non-viable sperm in the ejaculate: lethal escorts for contemporary viable sperm. Anim Reprod Sci.

[6] Yi YJ, Zimmerman SW, Manandhar G, Odhiambo JF, Kennedy C, Jonáková V (2012). Ubiquitin-activating enzyme (UBA1) is required for sperm capacitation, acrosomal exocytosis and sperm-egg coat penetration during porcine fertilization. Int J Androl.

[7] Odhiambo JF, Sutovsky M, DeJarnette JM, Marshall C, Sutovsky P (2011). Adaptation of ubiquitin-PNA based sperm quality assay for semen evaluation by a conventional flow cytometer and a dedicated platform for flow cytometric semen analysis. Theriogenology.

[8] Durfey CL, Swistek SE, Liao SF, Crenshaw MA, Clemente HJ, Thirumalai RVKG (2019). Nanotechnology-based approach for safer enrichment of semen with best spermatozoa. J Anim Sci Biotechnol.

[9] Durfey CL, Burnetta DD, Liaoa SF, Steadmana CS, Crenshawa MA, Clemente HJ (2017). Nanotechnology-based selection of boar spermatozoa: growth development and health assessments of produced offspring. Livest Sci.

[10] Watson PF (1995). Recent developments and concepts in the cryopreservation of spermatozoa and the assessment of their post-thawing function. Reprod Fertil Dev.

[11] Bailey JL, Bilodeau J, Cormier N (2000). Semen cryopreservation in domestic animals: a damaging and capacitating phenomenon. J Androl.

[12] Yeste M (2016). Sperm cryopreservation update: Cryodamage, markers, and factors affecting the sperm freezability in pigs. Theriogenology.

[13] Walters EM, Benson JD, Woods EJ, Critser JK. The history of sperm cryopreservation. In: Pacey AA, Tomlinson MJ, editors. Sperm banking: theory and practice. Cambridge, UK: Cambridge University Press; 2009. p. 1–10.

[14] Purdy PH, Graham JK (2004). Effect of cholesterol-loaded cyclodextrin on the cryosurvival of bull sperm. Cryobiology.

[15] Purdy PH, Graham JK (2004). Effect of adding cholesterol to bull sperm membranes on sperm capacitation, the acrosome reaction, and fertility. Biol Reprod.

[16] Takeda K, Uchiyama K, Kinukawa M, Tagami T, Kaneda M, Watanabe S (2015). Evaluation of sperm DNA damage in bulls by TUNEL assay as a parameter of semen quality. J Reprod Dev.

[17] Fraser LR, Abeydeera LR, Niwa K (1995). Ca(2+)-regulating mechanisms that modulate bull sperm capacitation and acrosomal exocytosis as determined by chlortetracycline analysis. Mol Reprod Dev.

[18] Kay VJ, Coutts JR, Robertson L (1994). Effects of pentoxifylline and progesterone on human sperm capacitation and acrosome reaction. Hum Reprod.

[19] Jayasena CN, Radia UK, Figueiredo M, Revill LF, Dimakopoulou A, Osagie M (2019). Reduced testicular steroidogenesis and increased semen oxidative stress in male partners as novel markers of recurrent miscarriage. Clin Chem.

[20] Zaferani M, Cheong SH, Abbaspourrad A (2018). Rheotaxis-based separation of sperm with progressive motility using a microfluidic corral system. Proc Natl Acad Sci U S A.

[21] Zaferani M, Palermo GD, Abbaspourrad A (2019). Structures of a microchannel impose fierce competition to select for highly motile sperm. Science Adv.

[22] Pedley TJ, Kessler JO (1992). Hydrodynamic phenomena in suspensions of swimming microorganisms. Ann Rev Fluid Mech.

[23] Miki K, Clapham DE (2013). Rheotaxis guides mammalian sperm. Curr Biol.

[24] Kantsler V, Dunkel J, Blayney M, Goldstein RE (2014). Rheotaxis facilitates upstream navigation of mammalian sperm cells. eLife.

[25] Friedrich BM, Riedel-Kruse IH, Howard J, Jülicher F (2010). High-precision tracking of sperm swimming fine structure provides strong test of resistive force theory. J Exp Biol.

[26] Gaffney EA, Gadelha H, Smith DJ, Blake JR, Kirkman-Brown JC (2011). Mammalian sperm motility: observation and theory. Annu Rev Fluid Mech.

[27] Nosrati R, Driouchi A, Yip CM, Sinton D (2015). Two-dimensional slither swimming of sperm within a micrometer of a surface. Nat Commun.

[28] Tung CK, Ardon F, Roy A, Koch DL, Suarez SS, Wu M (2015). Emergence of upstream swimming via a hydrodynamic transition. Phys Rev Lett.

[29] David G, Serres C, Jouannet P (1981). Kinematics of human spermatozoa. Gamete Res.

[30] Eisenbach M, Giogalas LC (2006). Sperm guidance in mammals-an unpaved road to the egg. Nat Rev Mol Cell Biol.

[31] Bahat A, Eisenbach M (2006). Sperm thermotaxis. Mol Cell Endocrinol.

[32] Pérez-Cerezales S, Boryshpolets S, Afanzar O, Brandis A, Nevo R, Kiss V, Eisenbach M (2015). Involvement of opsins in mammalian sperm thermotaxis. Sci Rep.

[33] Bahat A, Tur-Kaspa I, Gakamsky A, Giojalas LC, Breitbart H, Eisenbach M (2003). Thermotaxis of mammalian sperm cells: a potential navigation mechanism in the female genital tract. Nat Med.

[34] Mondal MA, Takagi Y, Baba SA, Hamano KI (2017). Involvement of calcium channels and intracellular calcium in bull sperm thermotaxis. J Reprod Dev.

[35] Verstegen J, Iguer-Ouada M, Onclin K (2002). Computer assisted semen analyzers in andrology research and veterinary practice. Theriogenology.

[36] Garner DL, Thomas CA, Joerg HW, DeJarnette JM, Marshall CE (1997). Fluorometric assessments of mitochondrial function and viability in cryopreserved bovine spermatozoa. Biol Reprod.

[37] Amaral A, Lourenço B, Marques M, Ramalho-Santos J (2013). Mitochondria functionality and sperm quality. Reproduction.

[38] Küpker W, Schulze W, Diedrich K (1998). Ultrastructure of gametes and intracytoplasmic sperm injection: the significance of sperm morphology. Hum Reprod.

[39] Hirata S, Hoshi K, Shoda T, Mabuchi T (2002). Spermatozoon and mitochondrial DNA. Rep Med Biol.

[40] Sousa AP, Amaral A, Baptista M, Tavares R, Caballero Campo P, Caballero Peregrín P, Freitas A, Paiva A, Almeida-Santos T, Ramalho-Santos J (2011). Not all sperm are equal: functional mitochondria characterize a subpopulation of human sperm with better fertilization potential. PLoS One.

[41] Paoli D, Gallo M, Rizzo F, Baldi E, Francavilla S, Lenzi A, Lombardo F, Gandini L (2011). Mitochondrial membrane potential profile and its correlation with increasing sperm motility. Fertil Steril.

[42] Piomboni P, Focarelli R, Stendardi A, Ferramosca A, Zara V (2012). The role of mitochondria in energy production for human sperm motility. Int J Androl.

[43] Pelliccione F, Micillo A, Cordeschi G, D'Angeli A, Necozione S, Gandini L, Lenzi A, Francavilla F, Francavilla S (2011). Altered ultrastructure of mitochondrial membranes is strongly associated with unexplained asthenozoospermia. Fertil Steril.

[44] Ford WCL, Rees JM, Gagnon C (1999). The bioenergetics of mammalian sperm motility. Controls of sperm motility: biological and clinical aspects.

[45] Kasai T, Ogawa K, Mizuno K, Nagai S, Uchida Y, Ohta S, Fujie M, Suzuki K, Hirata S, Hoshi K (2002). Relationship between sperm mitochondrial membrane potential, sperm motility, and fertility potential. Asian J Androl.

[46] du Plessis SS, Agarwal A, Mohanty G, van der Linde M (2015). Oxidative phosphorylation versus glycolysis: what fuel do spermatozoa use?. Asian J Androl.

[47] Agarwal A, Sekhon LH (2010). The role of antioxidant therapy in the treatment of male infertility. Hum Fertil.

[48] López-Fernández C, Crespo F, Arroyo F, Fernández JL, Arana P, Johnston SD, Gosálvez J (2007). Dynamics of sperm DNA fragmentation in domestic animals II. The stallion. Theriogenology.

[49] Ballachey BE, Hohenboken WD, Evenson DP (1987). Heterogeneity of sperm nuclear chromatin structure and its relation to bull fertility. Biol Reprod.

[50] Erickson L, Kroetsch T, Anzar M (2015). Relationship between sperm apoptosis and bull fertility: in vivo and in vitro studies. Reprod Fertil Dev.

[51] Parrish JJ, Sko-Parrish J, Winer MA, First NL (1988). Capacitation of bovine sperm by heparin. Biol Reprod.

[52] Maxwell WM, Johnson LA (1997). Chlortetracycline analysis of boar spermatozoa after incubation, flow cytometric sorting, cooling, or cryopreservation. Mol Reprod Dev.

[53] Collin S, Sirard MA, Dufour M, Bailey JL (2000). Sperm calcium levels and chlorotetracycline fluorescence patterns are related to the in vivo fertility of cryopreserved bovine semen. J Androl.

[54] Dias EAR, Campanholi SP, Rossi GF, Freitas Dell'Aqua CP, Junior D’AJA, Papa FO, Zorzetto MF, CCP d P, Oliveira LZ, MEZ M, Monteiro FM (2018). Evaluation of cooling and freezing systems of bovine semen. Anim Reprod Sci.

[55] Shannon P, Vishwanath R (1995). The effect of optimal and suboptimal concentrations of sperm on the fertility of fresh and frozen bovine semen and a theoretical model to explain the fertility differences. Anim Reprood Sci.

[56] Sparr E, Halin L, Markova N, Wennerstrom H (2002). Phospholipids-cholesterol bilayers under osmotic stress. Biophys J.

[57] Srivastava N, Srivastava SK, Ghosh SK, Kumar A, Pamde M, Perumal P, Soni YK (2015). Cholesterol content of bull spermatozoa alters survival at ultra-low temperatures. Intl J Vet Sci Res.

