# Supplementary material for: Bovine sperm selection procedure prior to cryopreservation for improvement of post-thawed semen quality and fertility
Source: J Anim Sci Biotechnol. 2019 Nov 15;10:91. doi: 10.1186/s40104-019-0395-9 (PMC6857337; doi:10.1186/s40104-019-0395-9)
Supplement: Supplementary file 1 — Additional file 1. Detail information of cryopreservation. [file 40104_2019_395_MOESM1_ESM.pdf]

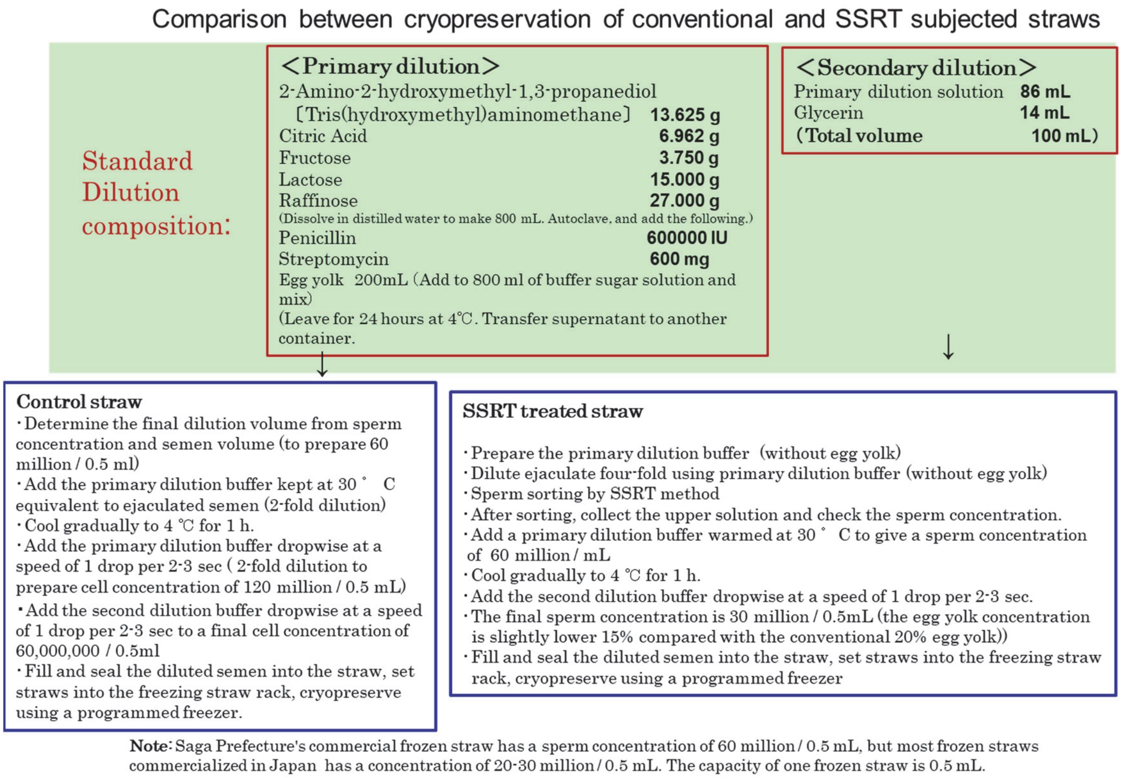

Figure S1. Cryopreservation of SSRT sorted and conventional unsorted (control) spermatozoa of bull ejaculates from 2 sires.
